# Study on the Permeability of Recycled Aggregate Pervious Concrete with Fibers

**DOI:** 10.3390/ma13020321

**Published:** 2020-01-10

**Authors:** Haitang Zhu, Chengcheng Wen, Zhanqiao Wang, Lan Li

**Affiliations:** 1 School of Civil Engineering, Henan University of Engineering, Zhengzhou 451191, China; htzhu@zzu.edu.cn; 2 School of Water Conservancy Engineering, Zhengzhou University, Zhengzhou 450001, China; wzqzzu@zzu.edu.cn; 3 Patent Examination Cooperation (Henan) Center of the Patent Office, CNIPA, Zhengzhou 450001, China; doraneverland@163.com

**Keywords:** recycled aggregate pervious concrete, permeability coefficient, water-cement ratio, flexural strength, porosity tortuosity

## Abstract

Pervious concrete is considered to be porous concrete because of its pore structure and excellent permeability. In general, larger porosity will increase the permeability coefficient, but will significantly decrease the compressive strength. The effects of water-cement ratio, fiber types, and fiber content on the permeability coefficient, porosity, compressive strength, and flexural strength were investigated. The pore tortuosity of the pervious concrete was determined by volumetric analysis and two-dimensional cross-sectional image analysis. The concept and calculation method of porosity tortuosity were further proposed. Results show that the permeability coefficient of the pervious concrete is the most suitable with a water-cement ratio of 0.30; the water permeability of the pervious concrete is influenced by fiber diameter. The permeability coefficient of pervious concrete with polypropylene thick fiber (PPTF) is greater than that with copper coated steel fiber (CCF) and the polypropylene fiber (PPF). The permeability coefficient is related to tortuosity and porosity, but when porosity is the same, the permeability coefficient may be different. Finally, general relations between the permeability coefficient and porosity tortuosity are constructed.

## 1. Introduction

Concrete is the most widely used material in civil infrastructure systems, and low strength concrete is usually used in earth rock dams, water-retaining walls, and other constructions [1,2]. It is especially used in the application of pervious concrete on non-structural pavement [3]. Pervious concrete is a porous lightweight concrete made of aggregate, cement, water, and admixture at a certain ratio. Permeability plays an important role in the performance characteristic of pervious concrete. Recent work has shown that the permeability coefficient of pervious concrete is mainly affected by porosity, and the porosity also affects the compressive strength and flexural strength of the hardened concrete. At higher porosity ratios, the permeability coefficient is increased, but the compressive strength and flexural strength are decreased [4]; thus, it is necessary to optimize the porosity in order to gain the desired strength and permeability coefficient.

According to Marinkovi et al. [5,6], waste concrete can be used as concrete aggregate after being crushed, sieved, and processed. Erhan Guneyisi [7] reported that adding recycled aggregate to pervious concrete can improve its permeability. Pervious concrete is widely used in pavement engineering, and it has the advantages of reducing noise, reducing water accumulation, improving the environment, and supplementing groundwater sources [8,9]. It has strong gas permeability, the porosity is usually in the range of 10% to 35%, the permeability ranges from 1.3 to 12.2 mm/s, and the density is 1600 to 2000 kg/m^3^ [10]. Because of its good drainage and osmosis system inside, pervious concrete can infiltrate rainwater during precipitation, making soil wet, so that the water source can be replenished downwards. In addition, the permeable rainwater can be slowly evaporated to relieve the heat island effect [11]. Usually it is promoted as a non-load bearing structure for parking lots and road surfaces.

Generally, compared with ordinary concrete, the strength and structural performance of pervious concrete is more variable because of the porosity [12]. A study by Haselbach [13] found that porosity varies not only with changes in the water-cement ratio and compressive strength, but also with changes in the pavement depth. This vertical pore distribution is caused by the surface compaction of the pervious concrete, which causes the top of the pavement to be denser than the bottom. Since larger porosity may lead to reduced strength, the distribution of vertical porosity may reduce the tensile strength at the bottom of the pavement. Because pervious concrete pavement is often damaged due to the formation of tensile cracks at the bottom, the flexural strength should be considered in the design of pervious concrete pavements.

Fibers have the effect of delaying and hindering cracks, and they are widely used in cement-based composite materials [14] and resin-based materials [15], which enhances the strength of the matrix. To further enhance the strength of pervious concrete, recent work by Hesami [16] indicated that polypropylene fibers can enhance the compressive, tensile, flexural, and abrasion strength, but they have no considerable effect on the modulus of elasticity of these specimens. Kevern [17] investigated the effect of synthetic fibers on the performance of pervious concrete. It was found that synthetic fibers reduced the permeability and abrasion resistance of it and increased the durability of freeze-thaw cycles. Wu [18] indicated that the addition of waste fibers had no significant effect on the compressive strength of the recycled aggregate pervious concrete, but it can enhance the flexural by 43%.

Porosity is used to characterize pervious concrete, but not all porosity is effective for water to flow through. Some pores are closed and do not connected with others. In addition, there are small pores, like capillary pores, and they have no contribution to the transport of fluid. Nevertheless, these pores affect the strength and permeability coefficient of pervious concrete. A new method was proposed by Kia Alalea [19] to study the pore blockage and to determine the possibility of pore blockage. Also, some scholars propose variable relationships between the permeability coefficient and porosity, which can be seen in the Kozeny–Carman equation and the Katz–Thompson equation [20,21,22].

The natural aggregate was replaced by recycled aggregate and recycled fine aggregate, and fiber and ultra-fine silicon powder (SP) were added in this study. The effects of fiber types, including polypropylene thick fiber (PPTF), copper coated steel fiber (CCF) and polypropylene fiber (PPF) with different percentages of the permeability, porosity, compressive strength, and flexural strength of the pervious concrete, are studied. In addition, three water-cement ratios of 0.25, 0.30, and 0.35 are evaluated. This study is an attempt to propose the concept and calculation method of tortuosity based on the characteristics of pore structure. Also, the second degree equation of the permeability coefficient, porosity, and tortuosity is fitted, where R^2^ = 0.99. This research can further promote the understanding and application of pervious concrete.

## 2. Experimental Program

### 2.1. Materials

According to GB175-2007 [23], PO42.5 Portland cement was selected in this test, and its physical properties and chemical composition are shown in Table 1. Ultra-fine silicon powder (SP) according to GB/T 51003-2014 [24] was used for all the mineral admixtures, and its physical properties and chemical composition are also shown in Table 1.

**Figure 1:**
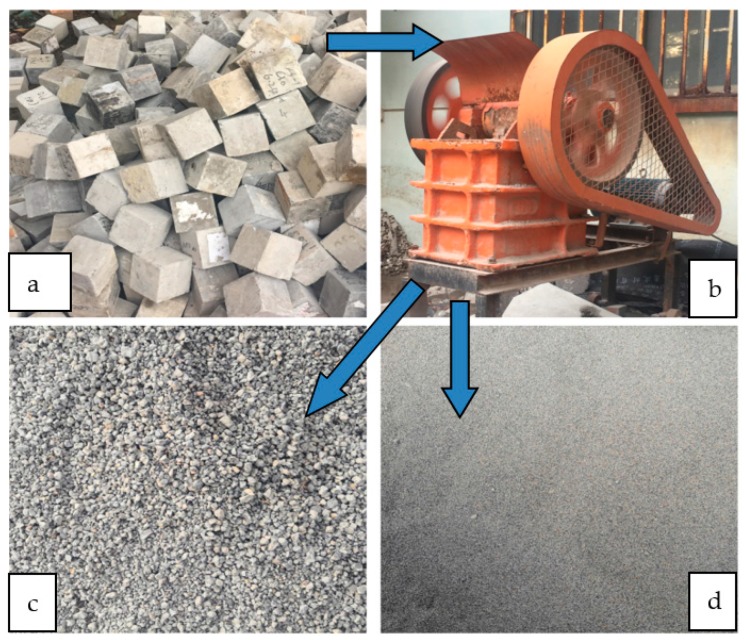
Preparation of recycled aggregate.

**Figure 2:**
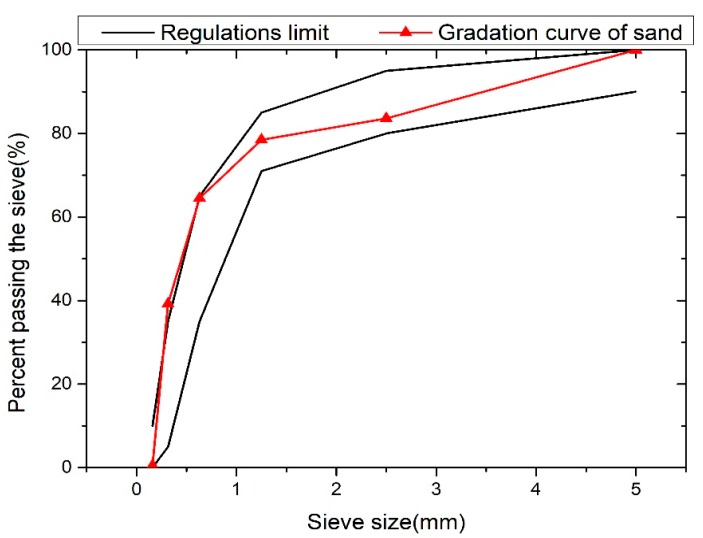
The particle size distribution curve of recycled fine aggregate.

As shown in Figure 1, the laboratory waste concrete (Figure 1a) was crushed in the jaw crusher (Figure 1b) to form 15–25 mm pieces; then, it was crushed by hammer crusher, and the recycled coarse aggregate (Figure 1c) of 5–10 mm particle sizes and the recycled fine aggregate (Figure 1d) of particle sizes less than 5 mm were screened. The physical properties of the recycled coarse aggregate are shown in Table 2. The recycled fine aggregate has a particle size of less than 5 mm and a fineness modulus of 2.7, which belongs to medium sand. The particle size distribution curve of the recycled fine aggregate is shown in Figure 2.

Three types of fibers were considered in the test. Their photos and characteristics are shown in Table 3.

### 2.2. Mixture Proportion

Generally speaking, according to ACI 522R 2010, the water-cement ratio is between 0.26 and 0.45 for pervious concrete and fine aggregates ranging from 0% to 15% [25]. CCF and PPF belong to short fibers, but CCF can effectively solve the corrosion of pervious concrete. Both PPF and PPTF are polypropylene materials, but the elastic modulus of PPTF is higher. The obtained mixture proportions are shown in Table 4.

### 2.3. Sample Preparation

Firstly, the aggregate, cement, and mineral admixture were added to the mixer, and then 80% water was added and it was stirred for 30 s to wet the cement into a cement slurry bonded to the aggregate surface. In the next step, the remaining water and fiber were then added together and stirred for a further 120 s to allow the cement to fully saturate and mix well with the fiber.

The cylinder specimens with a diameter of 100 mm and height of 50 mm were cast for the permeability coefficient and the porosity test; the cubic specimens with side length of 100 mm were cast for compressive strength test, and the prism specimens of 100 mm × 100 mm × 400 mm were cast for flexural strength test, in which the three specimens for each group were prepared.

The vibrating table was used firstly to vibrate the concrete, and then a trowel was applied to smooth the casting surface. After curing for 24 h in a natural environment, the specimens were transferred to a curing room at 95% RH and 20 °C for 28 days under standard conditions.

### 2.4. Experiment Methods

#### 2.4.1. Permeability Coefficient

**Figure 3:**
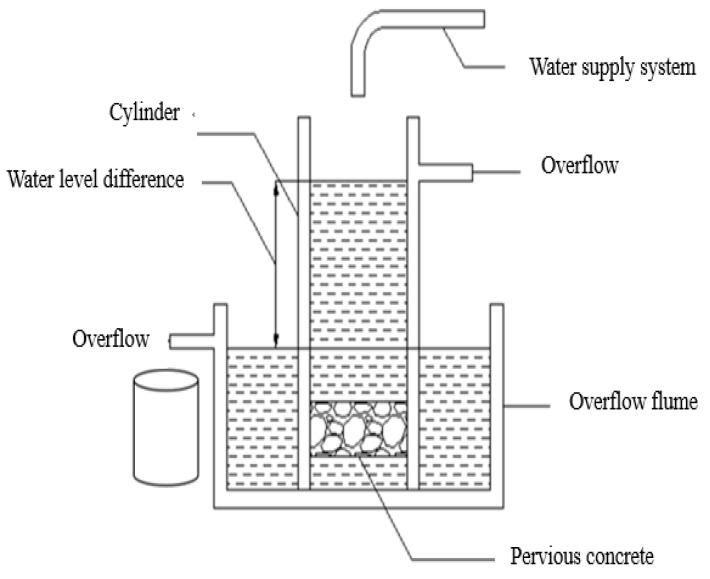
Schematic of permeability coefficient test set up.

The constant head method is derived from the Japan Society of Concrete Engineering, and its principle of measuring the permeability coefficient is Darcy’s law. The experiment device of the constant head method is shown in Figure 3.

The test specimen was sealed with a sealing material and placed on a bracket of the cylinder. After the water level difference was stabilized, the amount of overflow water *Q* in the time was measured again to calculate the permeability coefficient. The calculation of the permeability coefficient can be derived from the Darcy formula:

(1) Q=kAi

(2) i=H/L

(3) $$k=\frac{QL}{AHt}$$

where *k* is the permeability coefficient, *t* is the time, *Q* is the amount of overflow water in the time, *L* is the thickness of the test specimen, *A* is the surface area of the cylinder, *H* is the water level difference, *t* is the time, and *i* is the hydraulic slope.

#### 2.4.2. Volume Porosity

The test specimen was dried until it attained constant weight, and then we measured its apparent size. Next, the test specimen was immersed in a suitable container with water. Last, the container was shaken up and down to remove the air bubbles. The calculation formula for porosity is shown in Equation (4):

(4) $$P=1-\frac{V_{2}-V_{1}}{V}$$

where *P* is the porosity, *V* is the volume of test specimen after drying, *V*_1_ is the volume reading of the test specimen immersed in water, and *V*_2_ is the volume reading after the bubbles are discharged.

#### 2.4.3. Two-Dimensional Porosity

**Figure 4:**
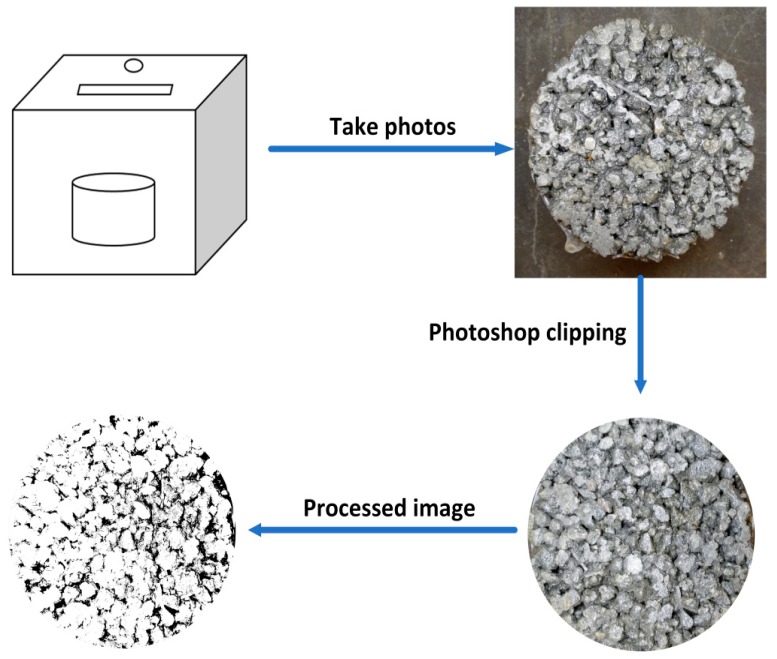
Collection and processing of two-dimensional image of pervious concrete.

As shown in Figure 4, the test specimen was placed in a box with good shading performance. In addition, a lamp was placed inside the box to provide lighting, and a hole was dug at the top of the box for photography. Each of the pervious concrete specimens was photographed on the front and back sides, and then we conducted black-and-white treatment of them by using Photoshop software. P’ means the average of two-dimensional porosity of the front and back of each test specimen.

## 3. Results and Discussions

Based on the methods and materials in the experiment, the main results of this test are shown in Table 5.

### 3.1. Effect of Water-Cement Ratio on Permeability

**Figure 5:**
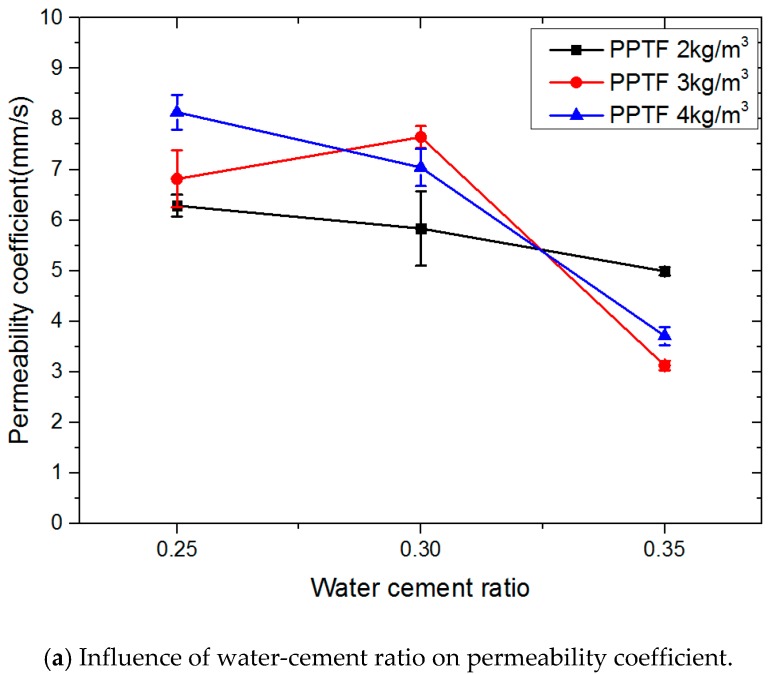

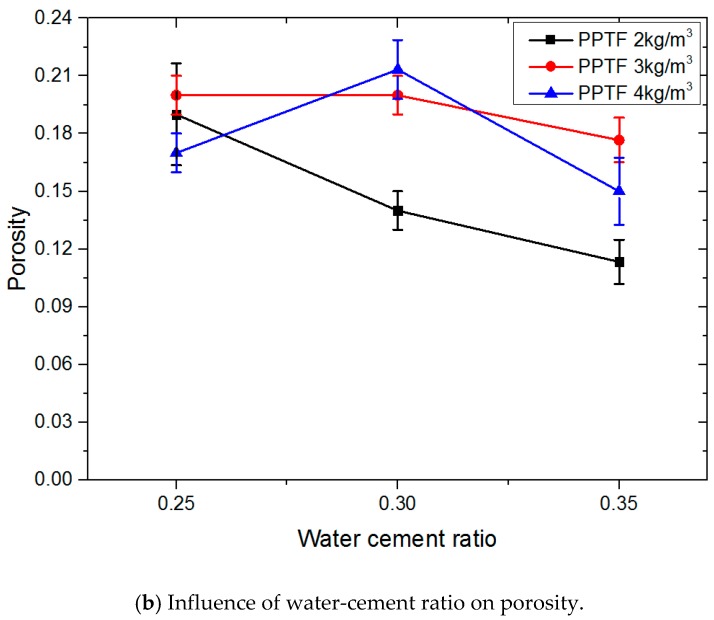
Influence of water-cement ratio on permeability of pervious concrete.

The effect of PPTF content on the water permeability of the pervious concrete at different water-cement ratios is shown in Figure 5. When the PPTF was 2 kg/m^3^, the permeability coefficient and porosity gradually reduced with the rise of water-cement ratio. When PPTF was 3 kg/m^3^, with the increase of the water-cement ratio, the permeability coefficient first increased by 12.02%, and then it decreased. The porosity decreased gradually, and the decreasing was minor. When the PPTF was 4 kg/m^3^, the permeability coefficient fell off gradually, while the porosity had a different trend: the figure for it climbed before dropping dramatically. The water-cement ratio of 0.30 was higher than the water-cement ratio by 0.25, with the number at 23.53%.

In general, the variation of porosity is slight while the range of permeability coefficients is pretty large. As the water-cement ratio raises, the permeability gradually reduces [26]. In fact, the influence of water-cement ratio on permeability is mainly achieved by changing the work performance of pervious concrete. When the water-cement ratio is low, the fluidity is weak, and it is difficult for cement slurry to be evenly wrapped on the surface of coarse aggregate. When the water-cement ratio is high, the fluidity is better. Under the action of gravity, the slurry stays at the bottom, improving the number of closed pores, which reduces the permeability of pervious concrete.

### 3.2. Effect of Fiber Types and Contents on Permeability

**Figure 6:**
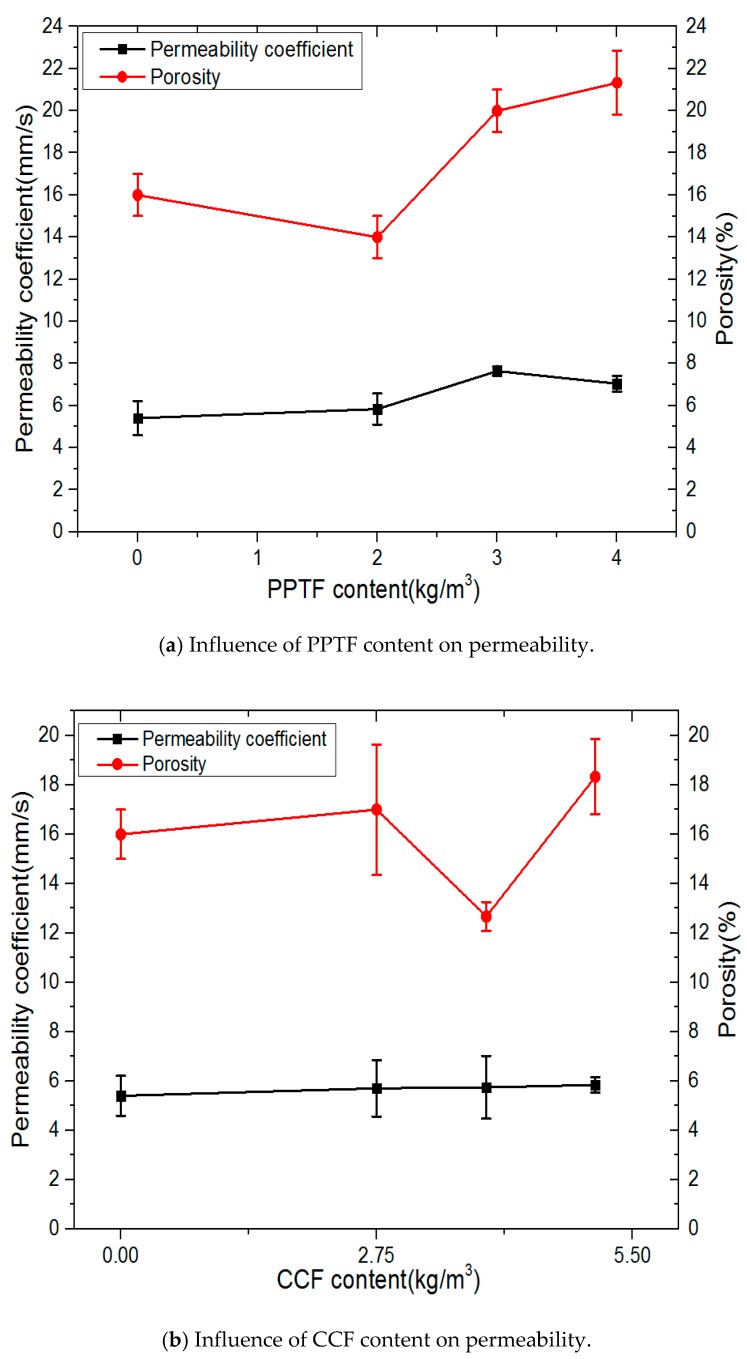

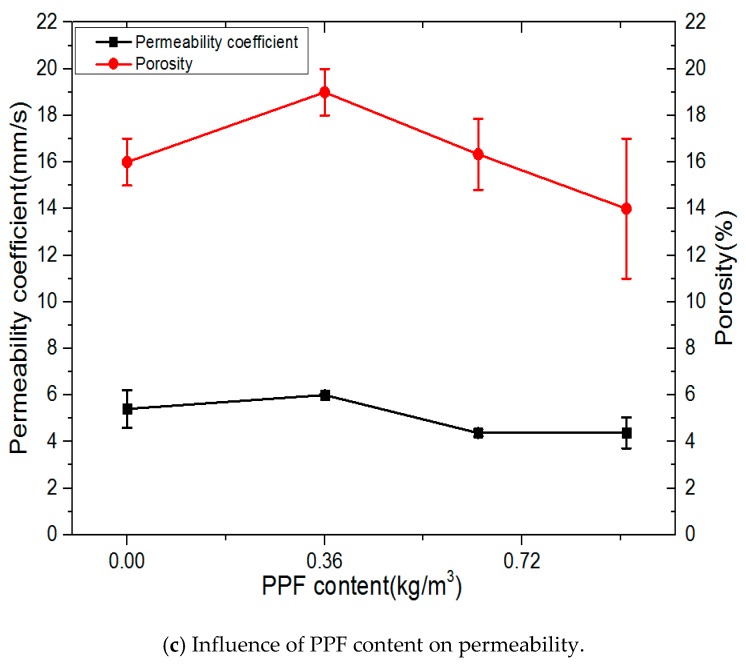
Influence of fiber type and content on permeability of pervious concrete.

It can be seen from the Figure 6 that in a water-cement ratio of 0.30, as was used for the permeability coefficient, the pervious concrete with PPTF enjoyed the best quality, while the pervious concrete for CCF ranked second, which is better than that for PPF.

As shown in Figure 6, with the raise of PPTF content, the permeability coefficient was boosted at first, and later the date of it dropped. However, a reverse trend was shown in the porosity, and its overall variation range was not obvious. As CCF content increased, the permeability coefficient inflated gradually and the amplitude was slight. When the fiber content was 3.93kg/m^3^, the porosity decreased rapidly. With the increase of PPF content, the porosity dropped after boosting. As for the permeability coefficient, there was an increase in the initial stage, but it suffered a reduction later and finally stabilized. Ali A. Aliabdo and H. Toutanji [27,28] also got similar results.

Because the porosity of the recycled aggregate pervious concrete is mainly composed of connected porosity and semi-connected pores, as fiber content increases, PPTF and CCF occupy a part of the volume of the pervious concrete connected pores, resulting in a decrease in connected porosity. However, the adhesion between fiber and cement substrate is relatively poor. Under the action of pressure, water can easily enter the interface between fiber and substrate, causing the interfaces to communicate with each other, so the connected porosity has an increase. In general, fiber content is low, and the water permeability coefficient varies little with the change in fiber content.

PPF has good dispersion in concrete, due to the incorporation of fibers, and the fluidity of the concrete is changed. As a result, the thickness distribution of the cement slurry between the aggregates is uneven, hence affecting the porosity of the recycled aggregate concrete. It can be known from physical properties of fiber that the diameter of PPF is the finest, CCF is the second, and the diameter of PPTF is the thickest. Therefore, it is apparent that the fiber diameter has a significant influence on the permeability of pervious concrete.

### 3.3. Effect of Water-Cement Ratio on Compressive Strength

**Figure 7:**
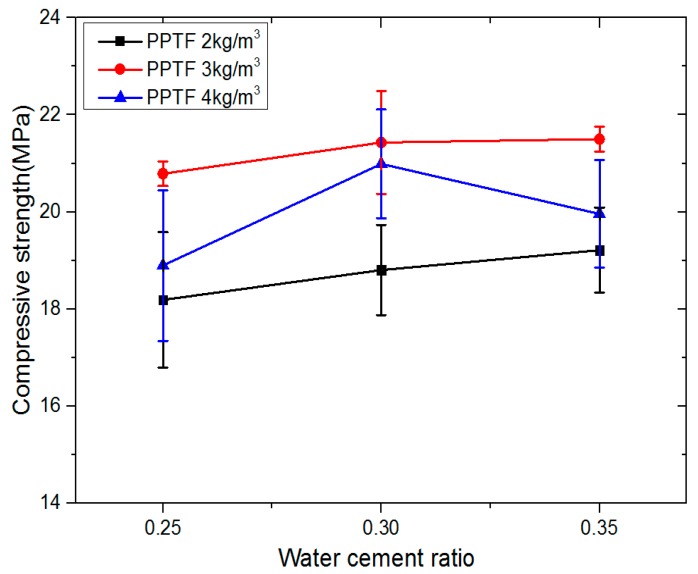
Influence of water-cement ratio on compressive strength.

The effect of the water-cement ratio on the compressive strength is shown in Figure 7. In the range of 0.25 to 0.35, the compressive strength rose first with the increase of the water-cement ratio, but then it reduced slightly. When the fiber content was 4 kg/m^3^, the compressive strength increased from 18.90 MPa to 20.99 MPa, and finally, reached 19.96 MPa, with a decrease of 8.94%. When the fiber content was 2 kg/m^3^, the compressive strength climbed slowly as the water ratio increased. For instance, as the water-cement ratio increased from 0.25 to 0.35, the compressive strength rose approximately 5.61%. In general, the compressive strength changes insignificantly as the water-cement ratio varies. This result is consistent with the test results of Saeid Hesami [29,30].

In the mix ratio, as the water-cement ratio increased, the amount of water increased and the amount of cement decreased. In the water-cement ratio of 0.25, the content of cement mortar was relatively low, and the aggregate could not be wrapped well, resulting in loose filling. Also, the hydration was insufficient, so the compressive strength of pervious concrete was eventually reduced.

In the water-cement ratio of 0.30, it had a certain fluidity, which improved the strength and bonding ability between the aggregates and the concrete saturate. The hydration was more sufficient, and the strength was higher than that of the pervious concrete with a water-cement ratio of 0.25. In the water-cement ratio of 0.35, the fluidity of mixture was so large that it could easily lead to a slurry. The strength of concrete structure was increased, but the lifting range was small. It can also be seen from the influence of water-cement ratio on porosity in Figure 5 that as the water-cement ratio rose, the porosity was gradually reduced, thus increasing the compressive strength, but the increasing scope was very small.

### 3.4. Effect of Fiber Types and Contents on Compressive Strength

**Figure 8:**
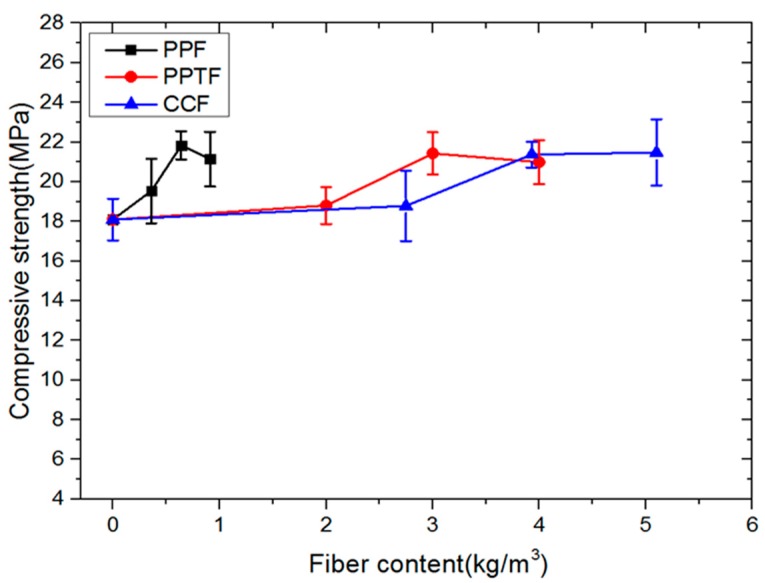
Influence of fiber types and content on compressive strength.

As shown in Figure 8, when no fiber was added, the compressive strength was 18.09 MPa. When the amount of fibers added increased, the compressive strength increased inconspicuously before a huge rise. When the number of PPTF content was 3 kg/m^3^, the compressive strength was the highest at 21.43 MPa. After that, there was a slight decrease. Generally speaking, CCF and PPF all contributed to raising the compressive strength. This finding agrees with the test results of Ali A. Aliabdo et al. [27] and Habulat et al. [31]. When the proper amount of fiber was added, the best compressive strength was gained.

PPF and PPTF have the same performance except for aspect ratio. However, the volume of PPF is significantly larger than that of PPTF, and the distribution is more random. CCF belongs to steel fiber, its compressive strength can reach 200 MPa, and it tends to go upward with the increase of fiber content. When the fiber content is too much, the dispersion is poor, and the interface between fiber and substrate is weak, which results in a decrease in the compressive strength of pervious concrete.

### 3.5. Effect of Fiber Types and Contents on Flexural Strength

**Figure 9:**
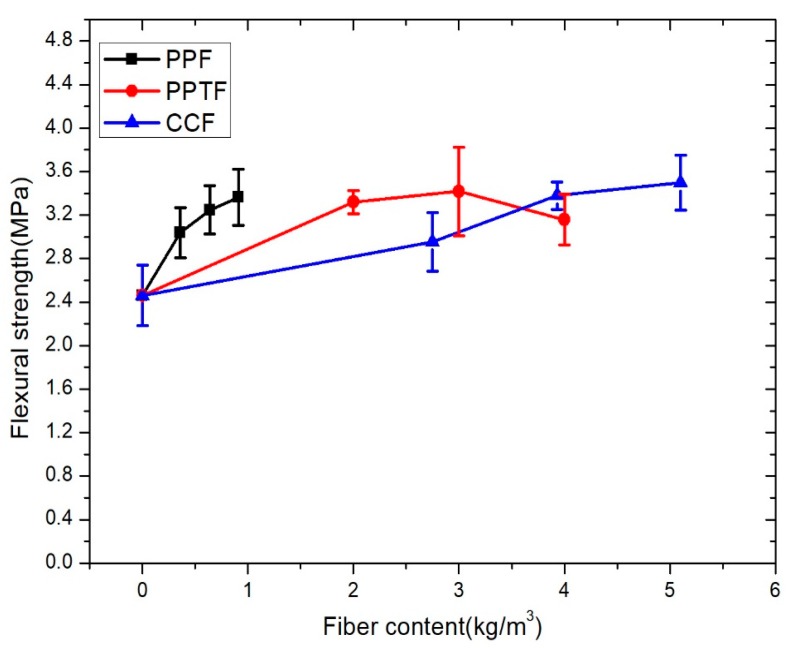
Influence of fiber types and content on flexural strength.

The influence of fiber types and content on flexural strength as shown in Figure 9, the incorporation of fibers had a high efficiency in improving the flexural strength of pervious concrete. PPTF had the highest flexural strength at 3.42 MPa, and compared with no fibers, the efficiency was 32.05%; the efficiency of CCF and PPF was 35.14% and 30.12%, respectively.

Three types of fibers all have contributed to improving flexural strength [32]. CCF and PPF had shorter lengths and fewer fibers at cracked sections. When they increase, they effectively participate in the flexural strength raised, which makes the improvement efficiency increase. PPTF is longer, and cement pastes cannot cover the PPTF fibers well. When too much PPTF is added, the efficiency of improving the flexural strength of permeable concrete shows a downward trend.

### 3.6. Permeability Calculation Model

#### 3.6.1. Pore Structure on Permeability

The permeability coefficient is an indicator that reflects the permeability of the pervious concrete. When designing the mix ratio of pervious concrete, the design porosity can be controlled to ensure the permeability coefficient. However, due to differences in raw material properties and differences between the concrete forming process and the pouring quality, the actual porosity of pervious concrete cannot be guaranteed to be the same as the designed porosity [33]. Moreover, the design porosity can only reflect the volume of pores in the pervious concrete. Even if the actual porosity of different pervious concrete is the same, their permeability coefficients may vary greatly [20]. The permeability coefficient depends on the volume of pores and the curvature shape of the channels.

**Figure 10:**
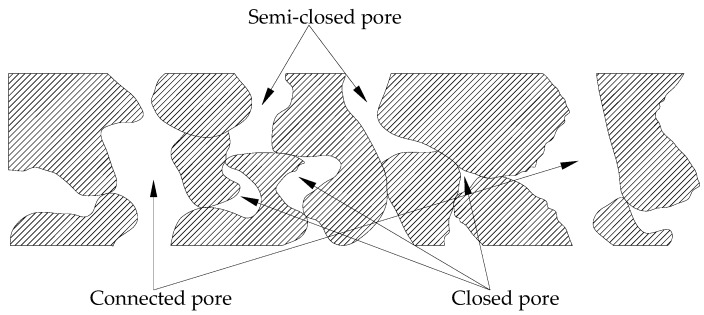
Pervious concrete pore types.

As shown in Figure 10, pores in pervious concrete are randomly distributed. There are connected pores forming channels, semi-closed pores, and even the pores that are closed and do not connect with others. Only the connected pores are permeable, and the semi-closed pores and closed pores do not contribute to concrete permeability [34]. In the existing evaluation system, the porosity and permeability coefficients can reflect the volume and overcurrent velocity of the pervious concrete. Tortuosity is introduced to reflect the degree of flow passage bending, and the evaluation system for permeability is a favorable supplement.

#### 3.6.2. Tortuosity

**Figure 11:**
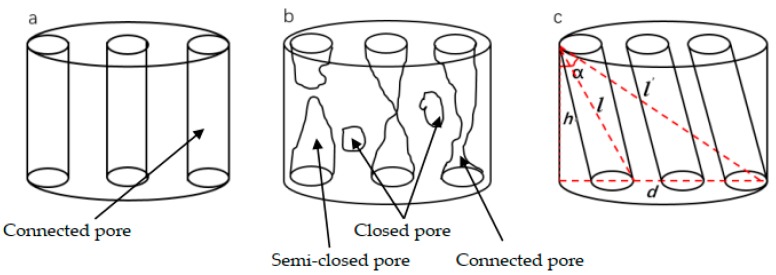
Pervious concrete percolation channels.

The percolation channels in pervious concrete are influenced by pore structure, mixture ratio, and the aggregate size. Ideal percolation channels are shown in Figure 11a, which shows the shortest process inside a pervious concrete specimen. Actually, there are various percolation channels, as shown in Figure 11b. Figure 11c is the simplified percolation channels of pervious concrete, showing the equivalent flow inside the pervious concrete specimen. The tortuosity is defined as the ratio R of the shortest flow length to the equivalent flow length. Intuitively, the geometrical meaning of the ratio of the shortest flow length to the equivalent flow length R is the cosine of the angle between the equivalent flow and the axis of the test piece.

In Figure 11c, the simplified triangle means the schematic diagram of the calculation of tortuosity where: *h* is the height of pervious concrete specimen (here 50 mm); *d* is the diameter of pervious concrete specimen (here 100 mm); α is the angle between the pervious concrete axis and the equivalent flow length. In the ideal state, the percolation channels of the permeable concrete are all connected pores with vertical distribution, *h* is the shortest percolation channels, and at this time α equals 0. In the extreme state, *l′* means the longest percolation channels in extreme state, and at this time α equals $\mathrm{arc}\cos\frac{1}{\sqrt{5}}$, so the value of cosα ranges from [$\frac{1}{\sqrt{5}}$,1].

The equivalent flow length *l* and the tortuosity R can be calculated by Formulas (5) and (6):

(5) $$l=\frac{\mathrm{PV}}{P{}^{\prime }A}=\frac{{\pi r}^{2}hP}{{\pi r}^{2}P{}^{\prime }}=\frac{hP}{P{}^{\prime }}$$

where P is the porosity, P’ is the average of two-dimensional porosity, A is the cross-sectional area, V is the volume of pervious concrete specimen, and *h* is the height of pervious concrete specimen.

(6) $$\cos a=R=\frac{h}{l}=\frac{P{}^{\prime }}{P}$$

where R is the tortuosity. The tortuosity calculation method is shown in Table 6.

**Figure 12:**
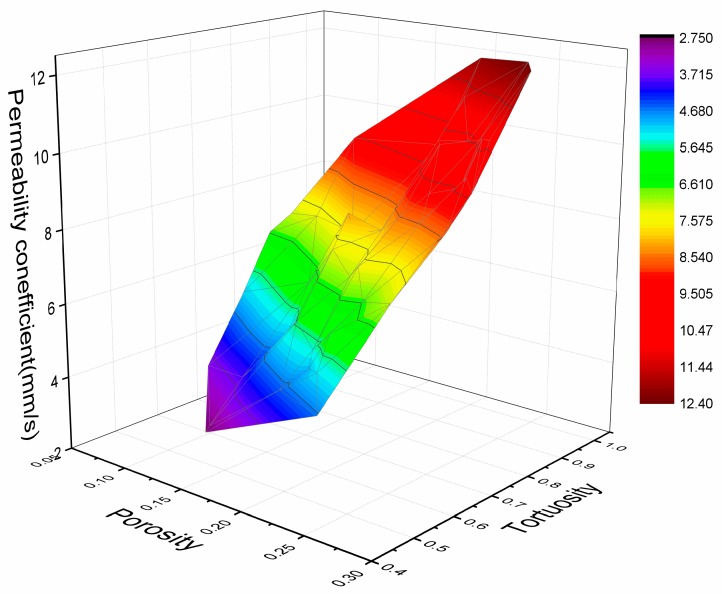
Influence of porosity and tortuosity on permeability coefficient.

Figure 12 shows the influence of porosity and tortuosity on the permeability coefficient. From Figure 12, the permeability coefficient *k* = 29.08*x*_1_ + 16.84*x*_2_ − 3.61*x*_1_*x*_2_ − 9.52, R^2^ = 0.99, where *k* is the permeability coefficient, *x*_1_ is the porosity, and *x*_2_ is the tortuosity. Table 6 shows the theoretical permeability coefficient and the actual permeability coefficient, and the results show that the error between the two is very tiny.

## 4. Conclusions

Based on the materials and measurement methods used in this test, the following conclusions can be drawn:

1. In a water-cement ratio of 0.30, the permeability of the recycled pervious concrete with fiber is the most appropriate. In a ratio of 0.25, the hydration is insufficient, while in the water-cement ratio of 0.35, excess cement slurry is easy to deposit at the bottom and seals the bottom pore.

2. For pervious concrete with different types of fibers, in the water-cement ratio of 0.30, the permeability coefficient of the recycled aggregate pervious concrete with PPTF is 7.64 mm/s, which is better than that of CCF and PPF at 5.74 mm/s and 6.01 mm/s, respectively.

3. In this paper, when the amount of PPTF content is 3 kg/m^3^, the permeability coefficient and strength have relatively balanced results. The flexural strength is the highest at 3.42 MPa, the compressive strength is 21.43 MPa, and the permeability coefficient is 7.64 mm/s.

4. The permeability coefficient of pervious concrete is linearly related to porosity, but the permeability coefficient may be different at the same porosity.

5. Fiber can significantly improve the flexural strength of pervious concrete. The flexural strength of pervious concrete is 2.46 MPa. When PPTF, CCF, and PPF were added, the flexural strength increased by 32.05%, 35.14%, and 30.12%, respectively.

6. The concept of tortuosity is proposed and a method for calculating tortuosity is provided. The equation of permeability coefficient, porosity, and tortuosity is fitted, where R^2^ = 0.99.

## Figures and Tables

**Table 1:** Physical properties and chemical composition of Portland cement and SP.

Material	Physical Properties		Chemical Composition							
	Density (g/cm^3^)	Surface Area (m^2^/kg)	SiO_2_ (%)	Al_2_O_3_ (%)	Fe_2_O_3_ (%)	CaO (%)	SO_3_ (%)	MgO (%)	K_2_O (%)	Na_2_O (%)
**Cement**	3.1	346	18.49	4.80	3.03	60.5	2.43	4.12	0.38	0.11
**SP**	2.6	1200	97.05	0.36	0.58	0.26	0.11	0.55	0.11	0.33

**Table 2:** Physical performance of recycled coarse aggregate.

Apparent Density (g/cm^3^)	Loose Packing Density (g/cm^3^)	Dry-Rodding Density (g/cm^3^)	Void Ratio (%)	Crush Index (%)	Water Absorption Rate (%)
2.412	1.264	1.429	44.17	89.5	3.896

**Table 3:** Photos and characteristics of fibers.

Fiber Type	PPTF	CCF	PPF
Fiber Photo	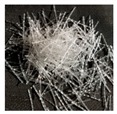	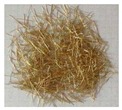	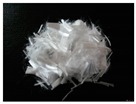
Mean Length(mm)	55	12	12
Mean Diameter (mm)	0.8	0.18	0.033
Density (g/cm^3^)	0.91	7.85	0.91
Main Characteristics	Elastic Modulus ≥ 5000 MPa	Flexural Strength ≥ 3000 MPa	Fracture Strength ≥ 300 MPa

**Table 4:** Mix proportions of pervious concrete.

Mix No.	Water-Cement Ratio	Fiber	Fiber Content (kg/m^3^)	Sand Rate	Cement (kg/m^3^)	SP (kg/m^3^)	Target Porosity
1	0.25	PPTF	2	10%	304.57	15.61	0.15
2	0.25	PPTF	3	10%	302.76	15.54	0.15
3	0.25	PPTF	4	10%	300.96	15.47	0.15
4	0.30	PPTF	2	10%	280.52	14.38	0.15
5	0.30	PPTF	3	10%	278.86	14.31	0.15
6	0.30	PPTF	4	10%	277.20	14.25	0.15
7	0.30	CCF	2.75	10%	279.28	14.33	0.15
8	0.30	CCF	3.93	10%	277.32	14.25	0.15
9	0.30	CCF	5.1	10%	275.37	14.17	0.15
10	0.30	PPF	0.36	10%	283.24	14.49	0.15
11	0.30	PPF	0.64	10%	282.79	14.47	0.15
12	0.30	PPF	0.91	10%	282.34	14.45	0.15
13	0.30	/	/	10%	283.85	14.51	0.15
14	0.35	PPTF	2	10%	260.00	13.33	0.15
15	0.35	PPTF	3	10%	258.46	13.27	0.15
16	0.35	PPTF	4	10%	256.92	13.20	0.15
17	0.30	PPTF	3	10%	357.81	17.57	0.10
18	0.30	PPTF	3	10%	199.91	11.15	0.20

**Table 5:** Summary of results.

No.	Porosity p	Permeability Coefficient k	Compressive Strength (MPa)	Flexural Strength (MPa)
1	0.19	6.29	18.19	2.56
2	0.20	6.82	20.79	2.93
3	0.17	8.12	18.90	3.03
4	0.14	5.84	18.80	3.32
5	0.20	7.64	21.43	3.42
6	0.21	7.04	20.99	3.16
7	0.17	5.71	18.78	2.86
8	0.13	5.74	21.38	3.38
9	0.18	5.85	21.48	3.5
10	0.19	6.01	19.54	3.04
11	0.16	4.38	21.84	3.25
12	0.14	4.38	21.15	3.37
13	0.16	5.58	18.09	2.46
14	0.11	4.99	19.21	3.02
15	0.18	3.13	21.50	3.56
16	0.15	3.71	19.96	3.22
17	0.13	5.58	18.65	2.91
18	0.18	5.74	18.12	2.7

**Table 6:** Tortuosity calculation method.

No.	Porosity P	Two-Dimensional Porosity $p_{1}^{\prime }$	Two-Dimensional Porosity $p_{2}^{\prime }$	Average Porosity p′	Tortuosity R	Actual Permeability Coefficient k	Theoretical Permeability Coefficient k′
5-1	0.28	19.46	19.63	19.55	0.70	9.62	9.70
5-2	0.21	14.45	13.76	14.11	0.65	7.40	7.04
5-3	0.24	16.82	17.16	16.99	0.71	8.59	8.80
5-4	0.20	13.25	13.35	13.30	0.66	7.02	6.93
5-5	0.22	15.88	16.16	16.02	0.72	8.52	8.43
5-6	0.15	9.85	9.87	9.86	0.65	4.96	5.44
5-7	0.13	7.02	6.94	6.98	0.52	2.92	2.77
5-8	0.15	10.64	10.47	10.56	0.71	6.28	6.41
5-9	0.17	11.03	10.76	10.90	0.62	5.73	5.48
5-10	0.13	7.92	7.65	7.79	0.60	4.08	4.08
5-11	0.13	6.78	6.81	6.80	0.51	2.78	2.61
5-12	0.19	10.35	9.97	10.16	0.52	4.51	4.41
5-13	0.07	5.15	5.03	5.09	0.77	5.11	5.29
5-14	0.08	5.12	5.26	5.19	0.65	3.53	3.56
5-15	0.20	11.54	11.23	11.39	0.57	5.45	5.48
17-1	0.22	13.11	12.26	12.69	0.55	6.13	5.70
17-2	0.22	12.85	14.03	13.44	0.63	6.63	6.99
17-3	0.22	12.02	11.53	11.78	0.52	5.25	5.22
17-4	0.17	12.82	13.05	12.94	0.76	7.63	7.76
17-5	0.11	9.20	9.20	9.20	0.85	7.36	7.66
17-6	0.17	13.62	12.94	13.28	0.75	8.19	7.59
17-7	0.13	7.47	8.05	7.76	0.60	3.73	4.08
17-8	0.12	10.98	11.08	11.03	0.92	9.44	9.06
17-9	0.21	20.08	20.31	20.20	0.97	12.02	12.19
17-10	0.20	10.68	11.13	10.91	0.56	4.99	5.32
17-11	0.27	20.77	21.47	21.12	0.78	10.64	10.71
17-12	0.24	10.98	10.86	10.92	0.46	4.63	4.81
17-13	0.17	12.68	12.22	12.45	0.71	7.34	6.94
17-14	0.07	5.79	5.86	5.83	0.84	6.63	6.45
17-15	0.08	7.60	7.20	7.40	0.87	7.16	7.21
18-1	0.26	18.50	19.11	18.81	0.74	9.49	9.81
18-2	0.28	24.75	24.10	24.43	0.85	12.26	12.08
18-3	0.24	18.15	17.33	17.74	0.73	9.25	9.12
18-4	0.27	20.36	19.68	20.02	0.72	9.88	9.75
18-5	0.27	23.62	24.03	23.83	0.88	12.38	12.29
18-6	0.27	20.77	20.88	20.83	0.76	10.73	10.39
18-7	0.24	18.91	19.50	19.21	0.83	10.31	10.72
18-8	0.26	15.06	14.58	14.82	0.57	7.17	7.10
18-9	0.23	19.00	18.49	18.75	0.79	10.27	9.82
18-10	0.20	14.79	15.01	14.90	0.74	7.97	8.22
18-11	0.22	20.05	19.97	20.01	0.89	11.20	11.16
18-12	0.22	17.97	18.22	18.10	0.82	10.24	10.04
18-13	0.27	17.36	17.99	17.68	0.67	8.64	8.96
18-14	0.21	17.27	17.72	17.50	0.86	9.96	10.42
18-15	0.26	20.48	20.26	20.37	0.79	10.57	10.60

## References

[1] Ma H., Chi F. (2016). Major Technologies for Safe Construction of High Earth-Rockfill Dams. Engineering.

[2] Ferdous W., Bai Y., Almutairi A.D., Satasivam S., Jeske J. (2018). Modular assembly of water-retaining walls using GFRP hollow profiles: Components and connection performance. Compos. Struct..

[3] Meininger Richard C. (1988). No-fines pervious concrete for paving. Concr. Int..

[4] Ghafoori N., Dutta S. (1995). Pavement thickness design for no-fines concrete parking lots. J. Trans. Eng..

[5] Marinkovic S., Dragas J., Ignjatovic I., Tosic N. (2017). Environmental assessment of green concretes for structural use. J. Clean. Prod..

[6] Xiao J.Z., Li W.G., Fan Y.H., Xiao H. (2012). An overview of study on recycled aggregate concrete in China (1996–2011). Constr. Build. Mater..

[7] Güneyisi E., Gesoğlu M., Kareem Q., İpek S. (2016). Effect of different substitution of natural aggregate by recycles aggregate on performance characteristics of pervious concrete. Mater. Struct..

[8] Tsang C., Shehata M.H., Lotfy A. (2016). Ptimizing a test method to evaluate resistance of pervious concrete to cycles of freezing and thawing in the presence of different deicing salts. Materials.

[9] Ho H.L., Huang R., Hwang L.C., Lin W.T., Hsu H.M. (2018). Waste-Based Pervious Concrete for Climate-Resilient Pavements. Materials.

[10] Tennis P.D., Leming M.L., Akers D.J. (2004). Pervious Concrete Pavements.

[11] Liu T.J., Wang Z.Z., Zou D.J. (2019). Strength enhancement of recycled aggregate pervious concrete using a cement paste redistribution method. Cem. Concr. Res..

[12] Crouch L.K., Cates M.A., Dotson V.J., Honeycutt K.R., Badoe D. (2003). Measuring the effective air void content of Portland cement pervious pavements. Cem. Concr. Aggreg..

[13] Haselbach L.H., Freeman R.M. (2007). Effectively estimating in situ porosity of pervious concrete from cores. J. ASTM Int..

[14] Lawler J.S., Zampini D. (2005). Microfiber and macrofiber hybrid fiber-reinforced concrete. J. Mater. Civ. Eng..

[15] Ferdous W., Ngo T.D., Nguyen K.T., Ghazlan A., Mendis P., Manalo A. (2018). Effect of fire-retardant ceram powder on the properties of phenolic-based GFRP composites. Compos. Part B.

[16] Geng Y., Wang Q., Wang Y., Zhang H. (2019). Influence of service time of recycled coarse aggregate on the mechanical properties of recycled aggregate concrete. Mater. Struct..

[17] Kevern J.T., Biddle D., Cao Q. (2015). Effects of Macrosynthetic Fibers on Pervious Concrete Properties. Am. Soc. Civ. Eng..

[18] Wu X., Zhou J., Kang T., Wang F., Ding X., Wang S. (2019). Laboratory investigation on the shrinkage cracking of waste fiber-reinforced recycled aggregate concrete. Materials.

[19] Kia A., Wong H.S., Cheeseman C.R. (2018). Defining clogging potential for pervious concrete. J. Environ. Manag..

[20] Neithalath N., Sumanasooriya M.S., Deo O. (2010). Characterizing pore volume, sizes, and connectivity in pervious concretes for permeability prediction. Mater. Charact..

[21] Zhong R., Wille K. (2016). Linking pore system characteristics to the compressive behavior of pervious concrete. Cem. Concr. Compos..

[22] Deo O., Sumanasooriya M., Neithalath N. (2010). Permeability reduction in pervious concretes due to clogging: Experiments and modeling. J. Mater. Civ. Eng..

[23] National Standard of the People’s Republic of China (2007). Common Portland Cement, GB175-2007.

[24] National Standard of the People’s Republic of China (2014). Technical Cord for Application of Mineral. Admixture, GB/T 51003-2014.

[25] ACI Committee 522 (2010). Report on Pervious Concrete, ACI 522R-10.

[26] Sonebi M., Bassuoni M.T. (2013). Investigating the effect of mixture design parameters on pervious concrete by statistical modelling. Constr. Build. Mater..

[27] Aliabdo A.A., Elmoaty AE M.A., Fawzy A.M. (2018). Experimental investigation on permeability indices and strength of modified pervious concrete with recycled concrete aggregate. Constr. Build. Mater..

[28] Toutanji H., McNeil S., Bayasi Z. (1998). Chloride permeability and impact resistance of polypropylene-fiber-reinforced silica fume concrete. Cem. Concr. Res..

[29] Ibrahim A., Mahmoud E., Yamin M., Patibandla V.C. (2014). Experimental study on Portland cement pervious concrete mechanical and hydrological properties. Constr. Build. Mater..

[30] Hesami S., Ahmadi S., Nematzadeh M. (2014). Effects of rice husk ash and fiber on mechanical properties of pervious concrete pavement. Constr. Build. Mater..

[31] Habulat A., Saman H.M., Sidek M.N.M., Hassan D., Hashim N.H. (2018). Strength development of pervious concrete embedded with latex and polypropylene fiber. Regional Conference on Science, Technology and Social Sciences (RCSTSS 2016).

[32] Nam J., Kim G., Yoo J., Choe G., Kim H., Choi H., Kim Y. (2016). Effectiveness of fiber reinforcement on the mechanical properties and shrinkage cracking of recycled fine aggregate concrete. Materials.

[33] Zhong R., Xu M., Netto R.V., Wille K. (2016). Influence of pore tortuosity on hydraulic conductivity of pervious concrete: Characterization and modeling. Constr. Build. Mater..

[34] Rehder B., Banh K., Neithalath N. (2014). Fracture behavior of pervious concretes: The effects of pore structure and fibers. Eng. Fract. Mech..

